# C-reactive protein deficiency ameliorates experimental abdominal aortic aneurysms

**DOI:** 10.3389/fimmu.2023.1233807

**Published:** 2023-09-11

**Authors:** Yu Fu, Haole Liu, Kexin Li, Panpan Wei, Naqash Alam, Jie Deng, Meng Li, Haibin Wu, Xue He, Haiwen Hou, Congcong Xia, Rong Wang, Weirong Wang, Liang Bai, Baohui Xu, Yankui Li, Yi Wu, Enqi Liu, Sihai Zhao

**Affiliations:** ^1^ Institute of Cardiovascular Science, Translational Medicine Institute, Xi’an Jiaotong University Health Science Center, Xi’an, Shaanxi, China; ^2^ Department of Cardiology, The Second Affiliated Hospital of Xi’an Jiaotong University, Xi’an, Shaanxi, China; ^3^ Department of Vascular Surgery, The Second Hospital of Tianjin Medical University, Tianjin, China; ^4^ Department of Surgery, Stanford University School of Medicine, Stanford, CA, United States; ^5^ Key Laboratory of Environment and Genes Related to Diseases, Ministry of Education, Xi’an, Shaanxi, China

**Keywords:** abdominal aortic aneurysms, C-reactive protein, macrophages, matrix metalloproteinase 2, inflammation

## Abstract

**Background:**

C-reactive protein (CRP) levels are elevated in patients with abdominal aortic aneurysms (AAA). However, it has not been investigated whether CRP contributes to AAA pathogenesis.

**Methods:**

CRP deficient and wild type (WT) male mice were subjected to AAA induction via transient intra-aortic infusion of porcine pancreatic elastase. AAAs were monitored by *in situ* measurements of maximal infrarenal aortic external diameters immediately prior to and 14 days following elastase infusion. Key AAA pathologies were assessed by histochemical and immunohistochemical staining procedures. The influence of CRP deficiency on macrophage activation was evaluated in peritoneal macrophages *in vitro*.

**Results:**

CRP protein levels were higher in aneurysmal than that in non-aneurysmal aortas. Aneurysmal aortic dilation was markedly suppressed in CRP deficient (aortic diameter: 1.08 ± 0.11 mm) as compared to WT (1.21 ± 0.08 mm) mice on day 14 after elastase infusion. More medial elastin was retained in CRP deficient than in WT elastase-infused mice. Macrophage accumulation was significantly less in aneurysmal aorta from CRP deficient than that from WT mice. Matrix metalloproteinase 2 expression was also attenuated in CRP deficient as compared to WT aneurysmal aortas. CRP deficiency had no recognizable influence on medial smooth muscle loss, lymphocyte accumulation, aneurysmal angiogenesis, and matrix metalloproteinase 9 expression. In *in vitro* assays, mRNA levels for tumor necrosis factor α and cyclooxygenase 2 were reduced in lipopolysaccharide activated peritoneal macrophages from CRP deficient as compared to wild type mice.

**Conclusion:**

CRP deficiency suppressed experimental AAAs by attenuating aneurysmal elastin destruction, macrophage accumulation and matrix metalloproteinase 2 expression.

## Introduction

Abdominal aortic aneurysms (AAA) are local dilation of aortic segments particularly infrarenal aorta resulted from media destruction and diagnosed when the diameter exceeds 50% of adjacent aortic diameter (1). AAAs progress asymptomatically but fatal upon premature rupture with an estimated annual death of 100,000 worldwide (1–3). While poorly defined, inflammation is crucial in AAA pathogenesis (1, 4).

C-reactive protein (CRP) is an acute inflammatory protein and increases dramatically in response to almost tissue injury, infection and inflammation, thus being used as an inflammatory marker in clinic (5–9). CRP binds phosphorylated choline of pneumococcal C-polysaccharides, activates classical complement pathway or interacts with the Fc gammaR1/2 (FcγR1/2) receptor, altogether promoting the clearance of pathogens and cellular debris (10). CRP also modulates the function of immune cells including macrophages and lymphocytes (11–15). Thus, CRP is important for both host defense and human disease pathogenesis by regulating innate and adaptive immunity.

In clinical AAAs, it has been reported that the CRP levels were positively associated with aneurysm diameter (16–20). CRP was also highly expressed in aneurysmal as compared to non-aneurysmal aortas (21). Additionally, serum CRP levels have been used for helping AAA diagnosis as well as predicting clinical outcomes following AAA repair (22, 23). However, it has not been investigated whether CRP mediates AAA pathogenesis. Therefore, this study assessed the influence of CRP deficiency on experimental AAA formation and progression in the intra-aortic elastase infusion-induced AAA model.

## Materials and methods

### Mice

CRP deficient mice were previously generated using CRISPR/Cas9 and homologous recombination technology to knock-in a STOP cassette at the ATG site of the CRP gene on C57BL/6 genetic background) at Shanghai Biomodel Organism Science & Technology Development Co., Ltd (Shanghai, China) (24). CRP deficient and C57BL/6 wild type (WT) mice were used for all experiments. The use and care of animals in this study were approved by the Laboratory Animal Management Committee of Xi’an Jiaotong University, Xi’an, China (No. 2022-623).

### Identification of CRP deficient mice

Genomic DNA was extracted from tail tips of less than 3 weeks old mice. Genotyping was conducted using the polymerase chain reaction (PCR) assay and gene-specific primer set. PCR primers were sense primer P1, (GCAGTTGGCCAGGGAAAGTT) and antisense primer P2 (CATGATCAGAAGGCACCAGAGTAG) for WT allele (PCR product size: 552 base pairs) and sense primer P1 and antisense primer P3 (CCTCGCCGGACACGCTGAA) for targeted allele (PCR product size: 637 base pairs), respectively. Quantitative real-time reverse transcription- polymerase chain reaction (qRT-PCR) was assessed CRP mRNA levels in liver tissues using the primers (**Table 1**). Western blotting analysis was utilized to determine CRP protein expression. Antibodies for Western blotting were a goat anti-mouse CRP polyclonal antibody (1:4000, Cat#: BAF1829, R&D Systems, Inc, Minneapolis, MN, USA), a rabbit anti-mouse β-actin polyclonal antibody (1:10000, Cat#: bs-0061R, Bioss Technology, Beijing, China), and an horseradish peroxidase-conjugated donkey anti-goat polyclonal antibody (1:5000, Cat#: EK030, Zhuangzhi Biological Technology, Xi’an, China) or goat anti-rabbit polyclonal antibody (1:10000, Cat#: bs-40295G, Bioss Technology) (25).

**Table 1:** Primer sequences used for qRT-PCR assay.

Gene	Forward (5’-3’)	Reverse (5’-3’)
C-reactive protein	GTC TGC TAC GGG GAT TGT AGA	CAC CGC CAT ACG AGT CCT G
Interleukin-1β	CGT GGA CCT TCC AGG ATG AG	CAT CTC GGA GCC TGT AGT GC
Interleulin-6	CGG CCT TCC CTA CTT CAC AA	TTC TGC AAG TGC ATC ATC GT
Cyclooxygenase 2	CTG ACC CCC AAG GCT CAA AT	TCC ATC CTT GAA AAG GCG CA
Tumor necrosis factor-α	TGA GCA CAG AAA GCA TGA TCC	GCC ATT TGG GAA CTT CTC ATC
Arginase 1	CTT GCG AGA CGT AGA CCC TG	CTT CCT TCC CAG CAG GTA GC
Resistin-like molecule α	CTG GGA TGA CTG CTA CTG GG	CAG TGG TCC AGT CAA CGA GTA
Chitinase 3-like 3	CCA GCA GAA GCT CTC CAG AAG	TCA GCT GGT AGG AAG ATC CCA
beta-actin	CAT CCG TAA AGA CCT CTA TGC CAA C	ATG GAG CCA CCG ATC CAC A

### AAA creation in mice

Male CRP-deficient and WT control mice at 9 weeks of age were used for experiments. AAAs were induced in infrarenal aorta under sterile condition using porcine pancreatic elastase (PPE) infusion method as previously described (26–28). Briefly, mice were anesthetized by 2% isoflurane inhalation, and a laparotomy was created to expose the infrarenal aorta. Mice were infused with PPE solution for 5 minutes (1.5 units/mL, Cat#: E-1250, Sigma-Aldrich, St Louis, MO, USA) through the aortotomy in temporarily controlled infrarenal aortic segment using a pressure pump (29). Thereafter, aortotomy and laparotomy were sequentially closed using 10-0 and 6-0 silk sutures, respectively. Mice were recovered, housed in individual cages, and monitored daily for morbidity and mortality.

### Measurements of aneurysmal aortic diameters

External infrarenal aortic diameters were measured *in situ* using a digital microscope. Briefly, infrarenal aortic segment was photographed immediately prior to (baseline) and 14 days following PPE. Maximal external infrarenal aortic diameters were determined using Motic Image Plus 3.0 ML software (Motic Electric Group Co., Ltd, Xiamen, China). An AAA was defined as a more than 50% increase in external diameter over the baseline level (29).

### Immunostaining of CRP in experimental aneurysmal aorta

Mice were euthanized by carbon dioxide inhalation 14 days following PPE infusion. Aortas were harvested, embedded in optical cutting temperature compound, sectioned (6 μm) and fixed with cold acetone. Infrarenal aortas from naïve WT mice were processed identically and served as non-aneurysmal controls. Frozen sections were stained with hematoxylin and eosin (H&E) and a standard streptavidin peroxidase immunohistochemical method for the assessment of morphological alterations and CRP protein expression, respectively. Reagents for CRP tissue immunostaining were a rabbit anti-mouse CRP polyclonal antibody (1:200, Cat#: bs-0155R) and normal rabbit IgG (1:200, Cat#: bs-0295P) from Bioss Technology. Other reagents were biotinylated goat anti-rabbit IgG antibody (1:400, Cat#: BA-1000) and AEC substrate kit (Cat#: SK-4200, Vector Laboratories, Inc, Newark, CA, USA) and streptavidin-peroxidase conjugate (1:200, Cat#: 016-030-084, Jackson ImmunoResearch, West Grove, PA, USA).

### Histological analysis of medial elastin degradation and smooth muscle cell depletion in aneurysmal aortas

H&E and Elastic van Gieson (EVG) staining were performed frozen aortic sections to assess general morphological changes and elastin contents, respectively. To assess SMC retention, frozen aortic sections were sequentially stained with a goat anti-SMC α-actin polyclonal antibody (1:200, Cat#: NB300-978, Novus Biologicals, Centennial, CO, USA), a biotinylated rabbit anti-goat IgG antibody (1:400, Cat#: BA-5000, Vector Laboratories, Inc) and streptavidin-peroxidase conjugate (1:200, Cat#: 016-030-084, Jackson ImmunoResearch), and staining was visualized using the AEC substrate kit (Vector Laboratories, Inc). Elastin fragmentation and SMC loss were scored as the grade I (mild) to IV (severe) using a histological grading system as reported previously (26–31).

### Immunohistochemical staining for leukocytes, angiogenesis and matrix metalloproteinases in aneurysmal aortas

A standard biotin-streptavidin-peroxidase method was used for all immunohistochemical staining. Reagents used in this study were monoclonal antibodies against CD68 (macrophages, 1:200, clone #: FA-11, Cat#: 137002), CD4 (CD4^+^ T cells, 1:200, clone #: GK1.5, Cat#: 100402), CD8 (CD8^+^ T cells, 1:200, clone #: 53-6.7, Cat#: 100702), B220 (B cells, 1:200, clone #: RA3-6B2, Cat#: 103202) and CD31 (1:200, clone#: 390, Cat#: 102402) (all above mentioned primary antibodies from Biolegend Inc, San Diego, CA, USA), goat anti-mouse polyclonal antibodies against MMP2 (1:200, Cat#: AF1488, R&D Systems) and MMP9 (1:200, Cat#: AF909, R&D Systems), a biotinylated goat anti-rat secondary antibody (1:400, Cat#: BA-9400, Vector Laboratories, Inc) or rabbit anti-goat IgG secondary antibody (1:400, Cat#: BA-5000, Vector Laboratories, Inc), and streptavidin-peroxidase conjugate (1:200, Cat#: 016-030-084, Jackson ImmunoResearch) (27–32). Macrophage accumulation was scored as the grade I to IV, and the densities of CD4^+^ T cells, CD8^+^ T cells, B220^+^ B cells and angiogenesis were quantified as the number of positively stained cells or neovessels per aortic cross section (ACS) (30). MMP expression levels were quantified as a positively stained area percentage of aortic wall using WinRood 6.5 image software, Mitani Co. Ltd., Tokyo, Japan.

### Measurements of macrophage activation *in vitro*

Primary macrophages were isolated from 8 weeks old WT and CRP deficient mice 3 days following intraperitoneal injection of 3% thioglycolate and suspended in RPMI-1640 medium containing 10% fetal bovine serum and 1% penicillin/streptomycin. Following 6 hours activation with lipopolysaccharide (LPS, 50 ng/mL, Cat#: L2630, Sigma-Aldrich) or incubation with vehicle at 37°C, 5% CO_2_ for 6 h, cells were harvested. For interleukin-4 (IL-4) stimulated experiment, macrophages were treated with IL-4 (10 ng/mL; Cat#: 214-14, PeproTech, Inc. Cranbury, NJ, USA) or vehicle for 16 hours. Total RNA was extracted with RNAiso Plus (Cat#: 9109), and complementary DNA was synthesized PrimeScript RT kit (Cat#: RR036A) (all from Takara Bio Inc, Kusatsu, Shiga, Japan) and amplified using RealStar Green Power mix (Cat#: A311-10; GenStar, Beijing, China) and gene-specific primers (**Table 1**). mRNA levels were quantitated as fold changes in relative to vehicle-treated macrophages.

### Statistical analysis

All statistical analyses were performed using the GraphPad software Version 9.0 (Boston, MA, USA). Data on continuous variables were presented as either mean ± standard deviation if normally distributed, and statistical significance was tested using Student’s t-test, or one, two–way ANOVA followed by two group comparison tests. Otherwise, data were given as median and interquartile range, and statistical significance was determined using nonparametric Mann-Whitney test. Statistical significance level was set at p<0.05.

## Results

### CRP protein is increased in experimental aneurysmal aortas

**Figure 1:**
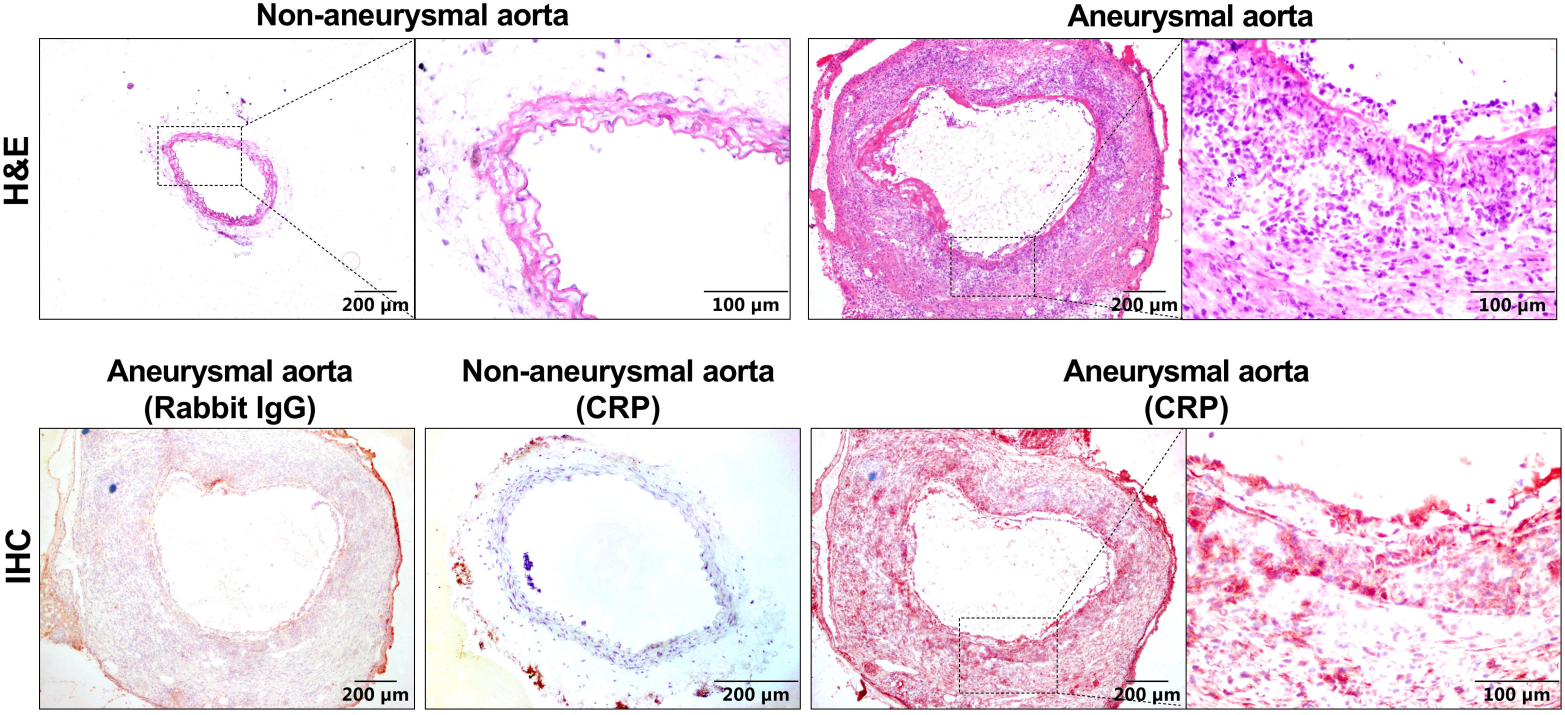
CRP expression is elevated in experimental aneurysmal aortas. H&E staining or CRP immunostaining using a rabbit anti-mouse CRP antibody or normal rabbit IgG as the negative control were performed on frozen sections from non-aneurysmal and aneurysmal aortas.

To examine whether CRP protein expression is altered in aneurysmal aortas, we stained aortic frozen sections from aneurysmal (PPE-infused) and non-aneurysmal WT mice. As depicted in **Figure 1**, rare or no positive staining was not seen in non-aneurysmal aorta. In contrast, an intense and diffusion CRP staining was noted in aneurysmal aortas (**Figure 1**). In serial sections, CRP staining was coincident with inflammatory cell accumulation in aneurysmal aortas.

### CRP deficiency mitigates experimental aneurysmal aortic dilation

**Figure 2:**
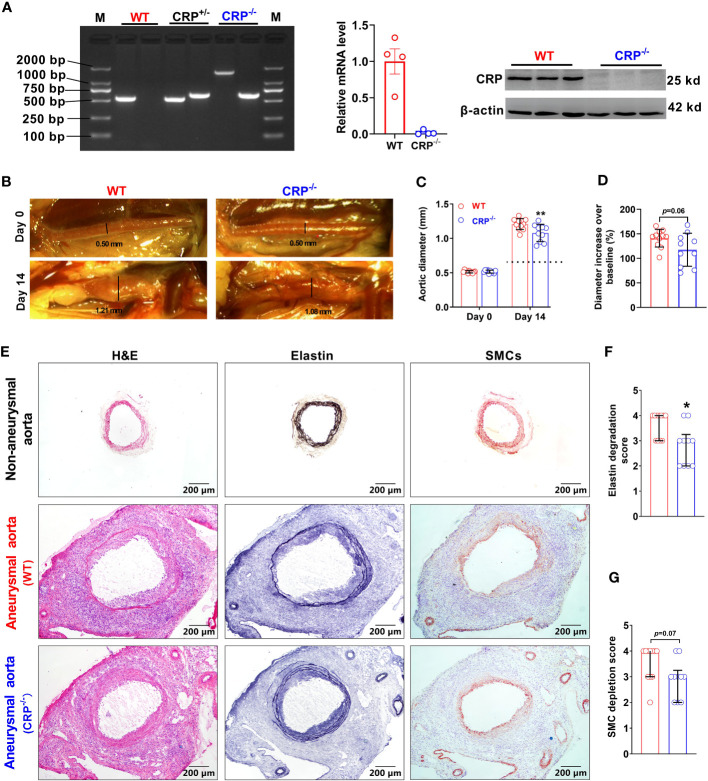
CRP deficiency suppresses experimental aneurysmal dilation. **(A)**: Phenotype identification of CRP deficient mice. Phenotyping for CRP homozygotes (CRP deficient mice: CRP^-/-^), CRP heterozygotes (CRP^+/-^) and wild type (WT) mice using PCR assay. CRP expression levels in the livers of CRP^-/-^ and WT mice were determined via qRT-PCR and Western blotting analyses. **(B)**: Representative photographic images for infrarenal aortas of wild type and CRP^-/-^ mice prior to and 14 days after the elastase infusion. AAAs were induced in male CRP^-/-^ and its wild type control mice using intra-aortic infusion of PPE. Influence on AAAs were assessed via *in situ* measurements of maximal infrarenal aortic diameters. Dotted line indicates aortic expansion even after PPE solvent (PBS) pressed infusion (about 0.8 mm in our lab) **(C, D)**: Maximal infrarenal aortic external diameters presented as absolute diameter on days 0 (baseline) and 14 after PPE infusion **(C)** or the percentage of diameters over baseline **(D)**. n=10-11 mice per group. Two-ANOVA followed by two group comparison, **p<0.01 compared to wild type mice at same timepoint **(C)**. Student *t*-test, p=0.06 compared to wild type mice **(D)**. **(E)**: Representative aortic images for H&E (left panels), elastin via Elastic Van Gieson (middle panels), and SMCs via an anti-SMC α antibody immunostaining (right panels) from non-aneurysmal and aneurysmal (wild type and CRP^-/-^) mice. **(F, G)**: Quantification of medial elastin degradation **(F)** and SMC depletion **(G)** scores (media and interquartile) of wild type and CRP^-/-^ aneurysmal aortas. Nonparametric Mann-Whitney test, *p<0.05 compared to wild type mice.

To clarify the effect of CRP in experimental AAAs, previously generated CRP-deficient (CRP^-/-^) mice were used in this study (24). The homozygotes of CRP-deficient mice were screened by PCR genotyping and Western blotting (**Figure 2A**). Luminal PPE infusion in infrarenal aorta was performed to induce AAAs in both WT and CRP^-/-^ mice. Fourteen days following PPE infusion, aortic dilation, as measured by external aortic diameter, was seen in both WT and CRP^-/-^ mice as compared to the baseline level. However, maximal external aortic diameter was significantly smaller in CRP^-/-^ (1.08 ± 0.11 mm) than that in WT (1.21 ± 0.08 mm) mice on day 14 after PPE infusion (**Figures 2B, C**). When subtracting aortic dilation due to the pressed infusion (approximately 0.8 mm), CRP deficiency per se led to a 32% reduction in aneurysmal enlargement (**Figure 2C**). Even considering the influence of baseline aortic diameter, a remarkable reduction in external aortic diameter was observed in CRP^-/-^ as compared to WT mice 14 days following PPE infusion (**Figure 2D**). Thus, CRP may in part mediate experimental aneurysmal expansion.

### CRP deficiency attenuates medial elastin degradation and SMC loss

In EVG staining for medial elastin, more medial elastin was maintained in CRP^-/-^ [score as 3 (2–3) (median with interquartile range)] as compared to WT [score as 4 (3–4)] mice (**Figures 2E, F**, p=0.019). Similarly, substantial retention of SMCs was noted in CRP^-/-^ [score as 3 (2–3)] as compared to WT [score as 4 (3–4)]. However, the difference in SMC grades did not reach statistical significant level (**Figures 2E, G**) probably due to insufficient sample size. Thus, experimental AAA suppression mediated by CRP deficiency is associated with marked preservation of medial elastin.

### CRP deficiency reduces macrophages accumulation in aneurysmal aorta

**Figure 3:**
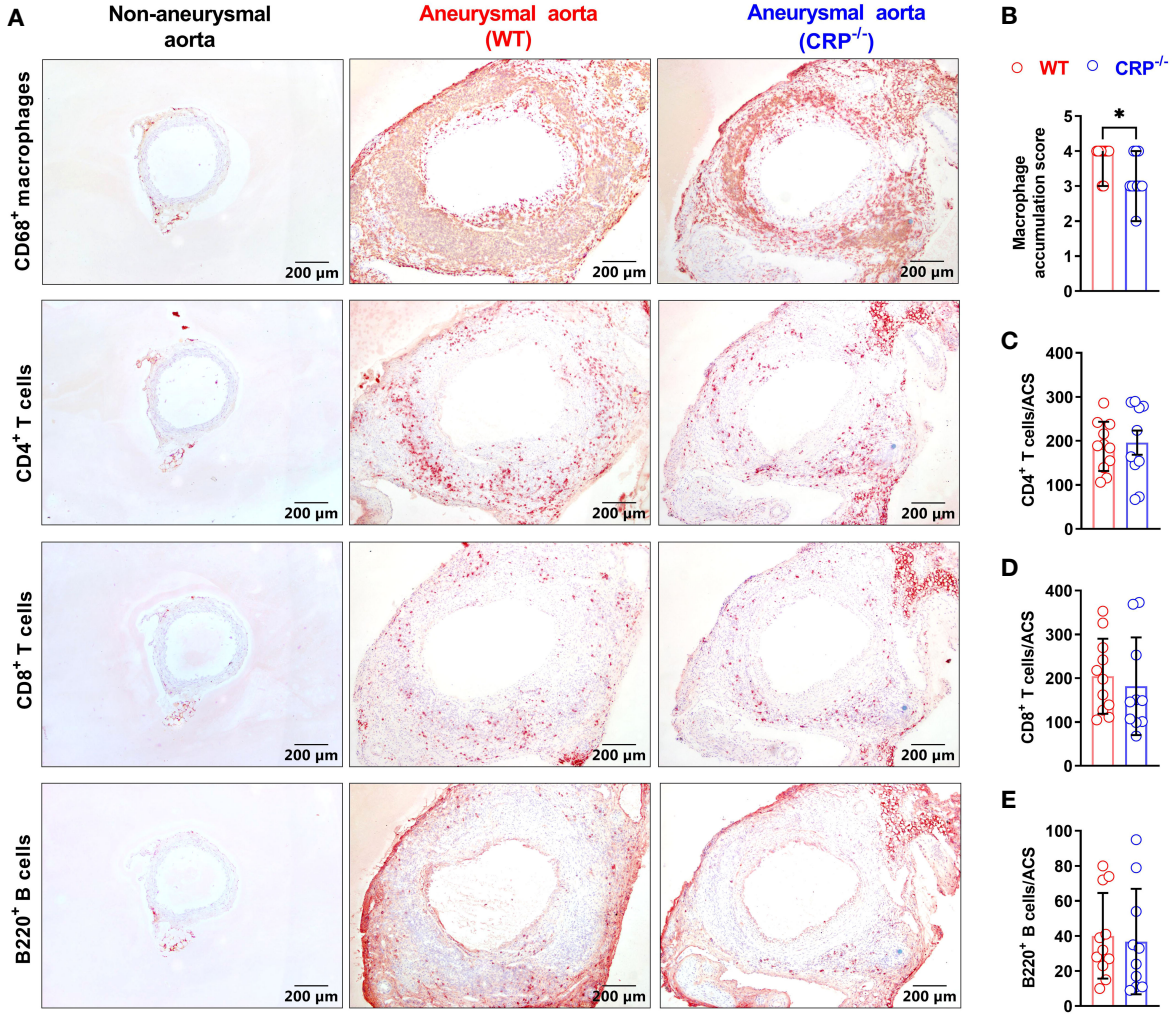
CRP deficiency alters aortic leukocyte accumulation. **(A)**: Representative aortic immunostaining images for CD68^+^ macrophages, CD4^+^ T cells, CD8^+^ T cells and B220^+^ B cells from non-aneurysmal normal aorta (left panels) and aneurysmal (wild type: middle panels; CRP^-/-^ mice: right panels). **(B)**: Quantification of macrophage accumulation scores (media and interquartile) in WT and CRP^-/-^ aneurysmal aorta. N=10-11 mice per group, Non-parametric Mann-Whitney, *P<0.01 compared to WT mice. **(C–E)**: Quantification of different lymphocyte subsets of aortic leukocytes [mean ± standard deviation for positive cells/aortic cross-section (ACS)] from the aneurysmal aortas of WT and CRP^-/-^ mice. No statistical difference in all lymphocyte subsets between two mouse strains.

Leukocytes contribute to experimental AAA pathogenesis (29, 33). In non-aneurysmal aorta, few leukocytes, almost CD68^+^ macrophages in aortic adventitia, were stained positively with subset-specific mAbs (**Figure 3A**). However, leukocytes infiltrated intensely throughout aortic wall in aneurysmal aortas (**Figure 3A**). CD68^+^ macrophages accumulated significantly less in CRP^-/-^ mice [score as 3 (2–4) (median with interquartile range)] than WT mice did [score as 4 (3–4)] (p=0.025) (**Figures 3A, B**). While CD4^+^ T cells, CD8^+^ T cells and B220^+^ B cells also accumulated in CRP^-/-^ and WT mice, no significant difference in either subset was seen between two mouse strains (**Figures 3A, C–E**).

### CRP deficiency diminishes MMP2 expression in aneurysmal aorta

**Figure 4:**
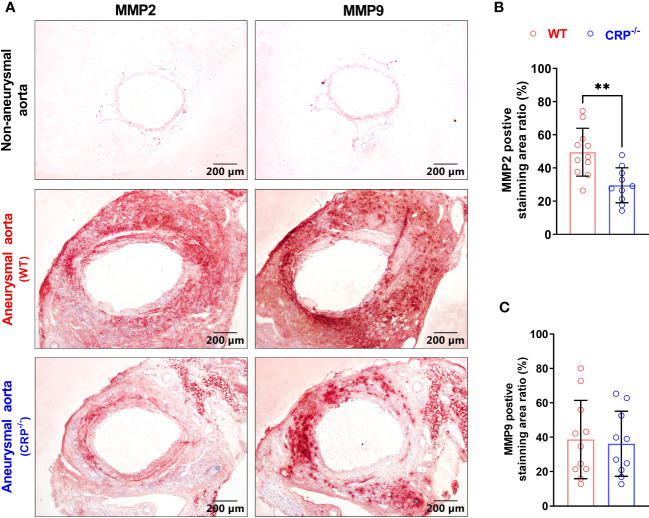
CRP deficiency reduces aneurysmal aortic MMP2 expression. **(A)**: Representative immunostaining images for MMP2 and MMP9 in non-aneurysmal normal or aneurysmal (WT and CRP^-/-^) aortas. **(B, C)**: Quantification of MMP2 and MMP9 expression (mean ± standard for the percentage of positively stained area in total aortic cross section area) in wild type and CRP^-/-^ aneurysmal aortas. Student’s *t* tests, n=10-11 mice per group, **p<0.01 compared to wild type mice.

Both MMP2 and MMP9 are important mediators of AAA pathogenesis (34). In our immunohistochemical staining, MMP2 expression was reduced in PPE-infused CRP^-/-^ [expressed as positive staining area ratio (%): 49.51 ± 13.77] as compared to WT (29.55 ± 9.98) mice (**Figure 4A**), with a 40% reduction in CRP^-/-^ mice (**Figure 4B**). However, there was no significant difference in MMP9 expression between PPE-infused CRP^-/-^ and WT mice (**Figures 4A, C**).

### CRP deficiency has no recognizable impact on angiogenesis in aneurysmal aorta

**Figure 5:**
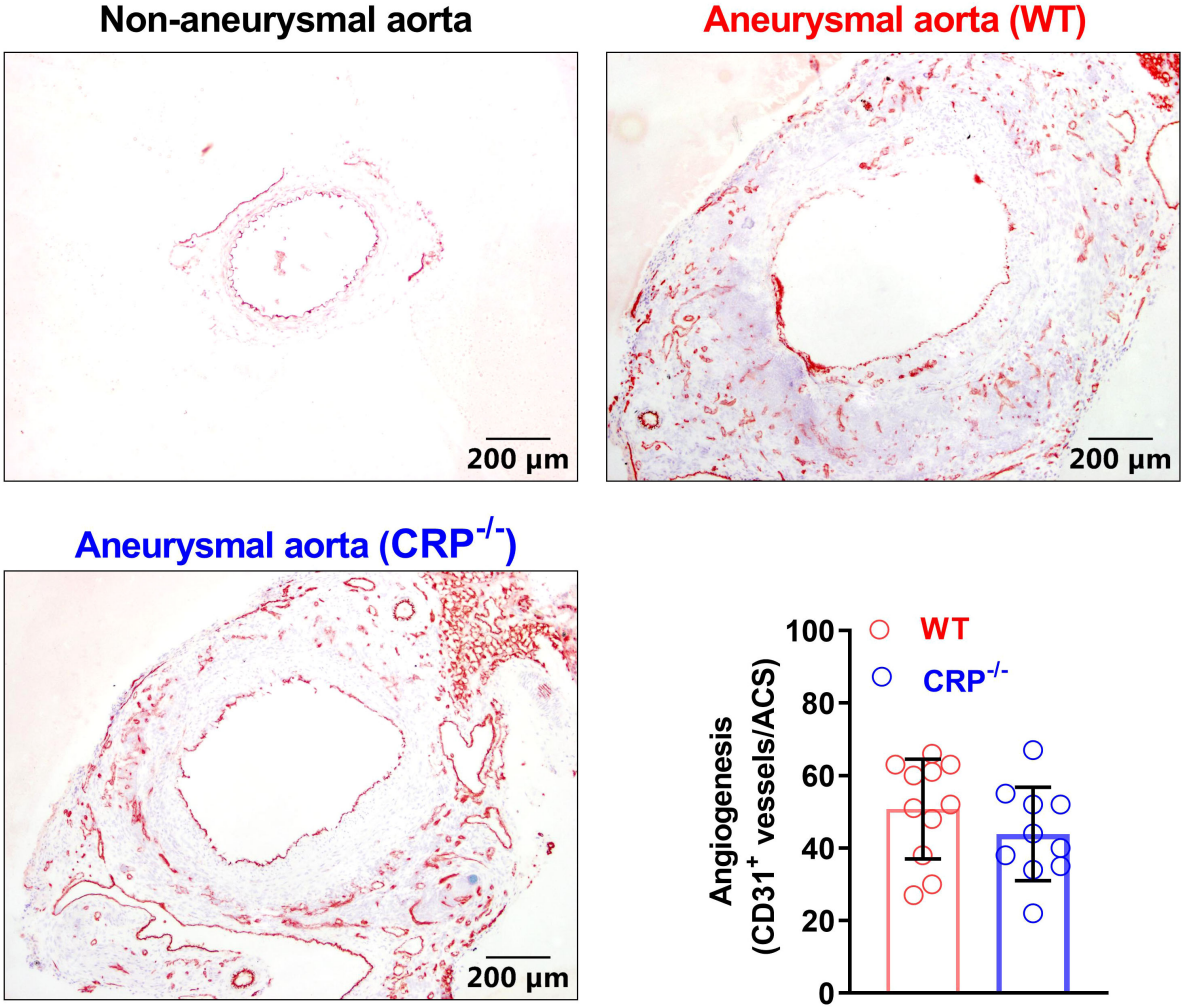
CRP deficiency has no remarkable impact on angiogenesis in aneurysmal aortas. Frozen sections were prepared from the aortas of non-aneurysmal normal (upper left) and aneurysmal (wild type: upper right; CRP^-/-^: lower left) mice (n=10-11 mice per group) and stained with an anti-CD31 antibody to assess aneurysmal angiogenesis. Lower right panel: angiogenesis in wild and CRP^-/-^ aneurysmal mouse aortas was quantitated as the number of CD31-positive neovessels per ACS. Data are mean ± standard deviation. Student’s *t* tests, no significant difference between two groups.

Angiogenesis, an additional key AAA pathology, also contributes to the progression of AAAs (35, 36). Angiogenesis, as determined by the density of CD31^+^ neovessels, was not differentiated between CRP^-/-^ (50.82 ± 13.40/ACS) and WT (43.90 ± 12.23/ACS) mice (**Figure 5**). Thus, neoangiogenesis may have no or limited contribution to the suppression of experimental AAAs by CRP deficiency.

### CRP deficiency limits classic macrophage activation *in vitro*

**Figure 6:**
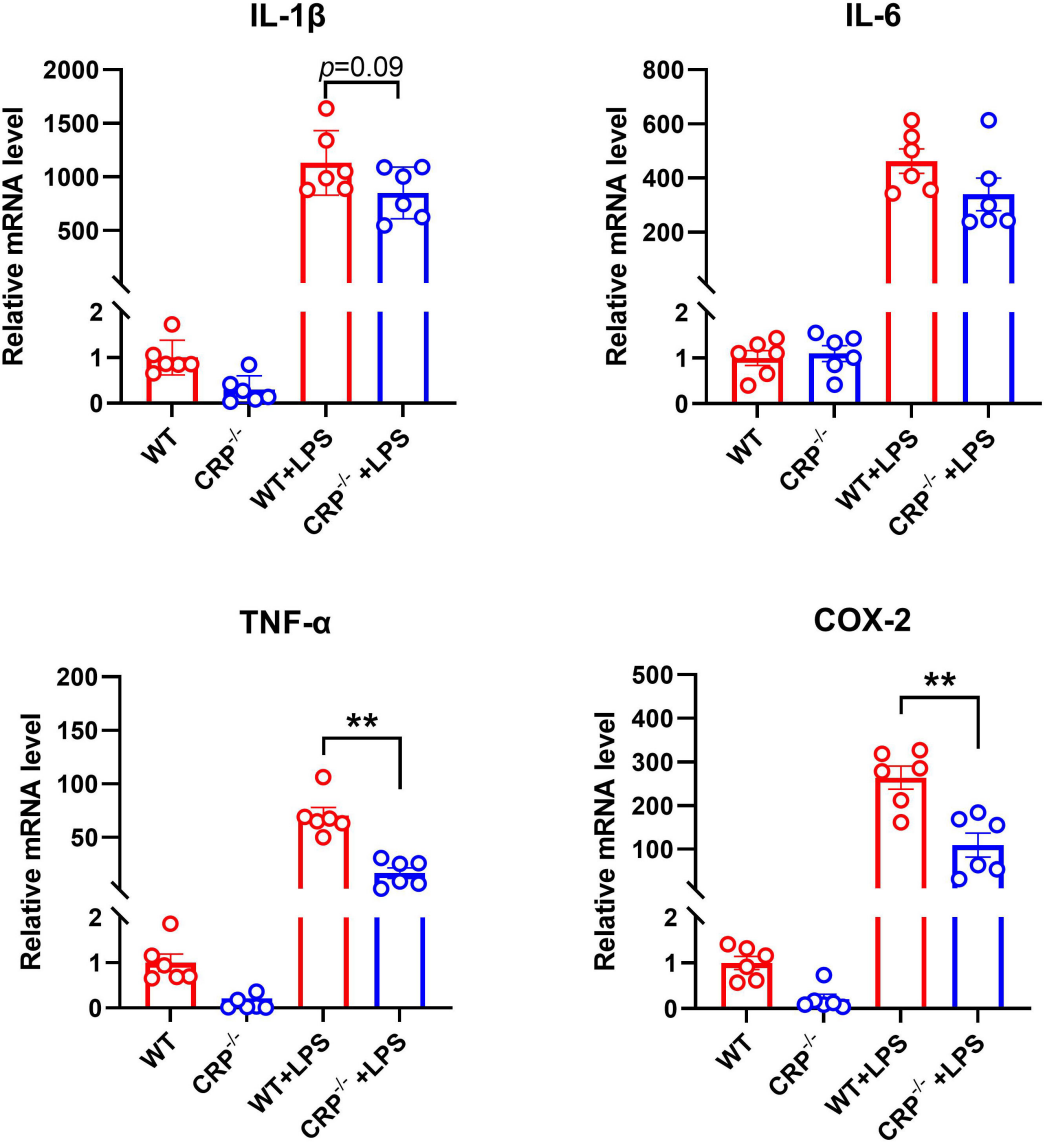
CRP deficiency impairs classic proinflammatory macrophage activation/polarization. Peritoneal macrophages from non-aneurysmal wild type (WT) and CRP^-/-^ mice underwent the classic activation (conventionally known as M1 macrophages) in the presence of LPS (50 ng/ml) or vehicle alone for 6 hours. The mRNA expression levels of indicated classic activation macrophage marker genes were detected by using qRT-PCR. mRNA levels are presented as mean ± standard deviation fold changes relative to vehicle-treated WT macrophages. Two-way ANOVA followed two group comparison, **p<0.01 between two groups, n=6 biological repeats for each group.

Classic macrophage activation (conventionally known as proinflammatory M1 macrophage activation/polarization) promotes AAA formation and progression (33, 37). In classical activated macrophage by LPS, the expression levels of mRNA for TNF-α and COX-2, but not IL-1β, IL-6 (M1 marker genes) were significantly reduced in CRP deficient as compared to WT macrophages (**Figure 6**). However, no significant influences were found for the expression levels of all alternative activation macrophage markers (conventionally knowns as anti-inflammatory M2 macrophages) after activated by IL-4 (**Supplementary Figure 1**).

## Discussion

Although the detailed mechanism of AAA pathogenesis is still unclear, the involvement of inflammation in the formation and progression of AAAs has become a consensus in this field (38). CRP, as one of the important inflammatory acute phase proteins, is involved in the development of many cardiovascular diseases (39–45). In this study, we found increased expression of CRP protein in experimental AAAs. CRP deficiency inhibited experimental AAA enlargement in the PPE infusion AAA model. Histologically, the suppression of experimental AAAs by CRP deficiency was associated with the attenuation of medial elastin destruction, aneurysmal wall macrophage accumulation and MMP2 expression. Additionally, CRP deficiency partially inhibited proinflammatory macrophage activation/polarization. Thus, our study indicated that CRP may in part mediate AAA pathogenesis.

In previous studies, CRP has been shown to regulate phenotypic differentiation and activity of macrophages (46–48). In patients with certain cardiovascular diseases, CRP expression levels was positively correlated with M1 macrophage activation (48). This was consistent with our findings that CRP deficiency downregulated the expression levels of M1 macrophages marker genes. Nuclear factor kappa B (NF-κB) regulates proinflammatory macrophages polarization as well as CRP activity, thus reduced M1 macrophage polarization due to CRP deficiency may potentially associate with altered NF-κB signaling activity (42, 47, 49, 50). Additionally, reduced M1 macrophage activation in CRP deficient macrophages may attenuate the expression of proaneurysmal mediators including cytokines and MMPs consequently leading to experimental AAA inhibition.

We found that CRP expression was elevated in experimental AAA lesion. While we have no data showing the source for increased CRP, this may result from locally and/or systemically increased CRP as reported in other pathological condition (51, 52). Macrophages have been reported to be the main cellular source for non-liver derived CRP (53). CRP interacts with macrophage FcγR1/2 or lectin-like oxidized LDL receptor 1 (LOX-1) and bind to Oxidized low-density lipoprotein (Ox-LDL), thus regulating macrophage functional activity through multiple pathways and mediating vascular inflammatory diseases (10, 54–57). Alternatively, the interaction of CRP with LOX-1 and Ox-LDL also enhances CRP expression in endothelial cells, which further promote vascular inflammation (58). Therefore, the potential modulation of macrophage activity by CRP may partially contribute to experimental AAA pathogenesis in the PPE AAA model.

Although the loss of endogenous CRP reduced experimental AAAs, the inhibition was not strong as demonstrated for other agents including metformin (59–61). Therefore, we need more experimental evidence to validate whether CRP can be a therapeutic target for AAA disease. In summary, this study demonstrates a partial role of CRP in experimental AAA pathogenesis of AAAs using CRP deficient mice.

## Data availability statement

The data presented in the study are included in the article/Supplementary Material. Further inquiries can be directed to the corresponding author.

## Ethics statement

The animal study was approved by Laboratory Animal Management Committee of Xi’an Jiaotong University. The study was conducted in accordance with the local legislation and institutional requirements.

## Author contributions

SZ, YW, EL, and BX designed this study; YF, HL, KL, SZ, JD, PW, ML, HW, XH, HH, RW, LB, WW, and YL collected and analyzed data, as well as drafted the manuscript; YF, HL, KL, PW, ML, HW, XH, and HH performed experiments; YW, NA, BX, and SZ critically revised manuscript. All authors contributed to the article and approved the submitted version.

## Supplementary material

Supplementary Figure 1 The effect of CRP deficiency in IL-4 induced M2 polarization in cultured macrophages. Peritoneal macrophages from non- WT and CRP^-/-^ mice underwent the alternative activation (conventionally known as M2 polarization) in the presence of IL-4 (10 ng/ml) or vehicle alone. The mRNA expression levels of indicated M2 macrophage marker genes were measured by using qRT-PCR. Two-way ANOVA followed two group comparison, no significant influence of CRP deficiency on either M2 maker mRNA levels, n=3 biological repeats for each group.
