# Supplementary figures and images for: C-reactive protein deficiency ameliorates experimental abdominal aortic aneurysms
Source: Front Immunol. 2023 Sep 11;14:1233807. doi: 10.3389/fimmu.2023.1233807 (PMC10518468; doi:10.3389/fimmu.2023.1233807)

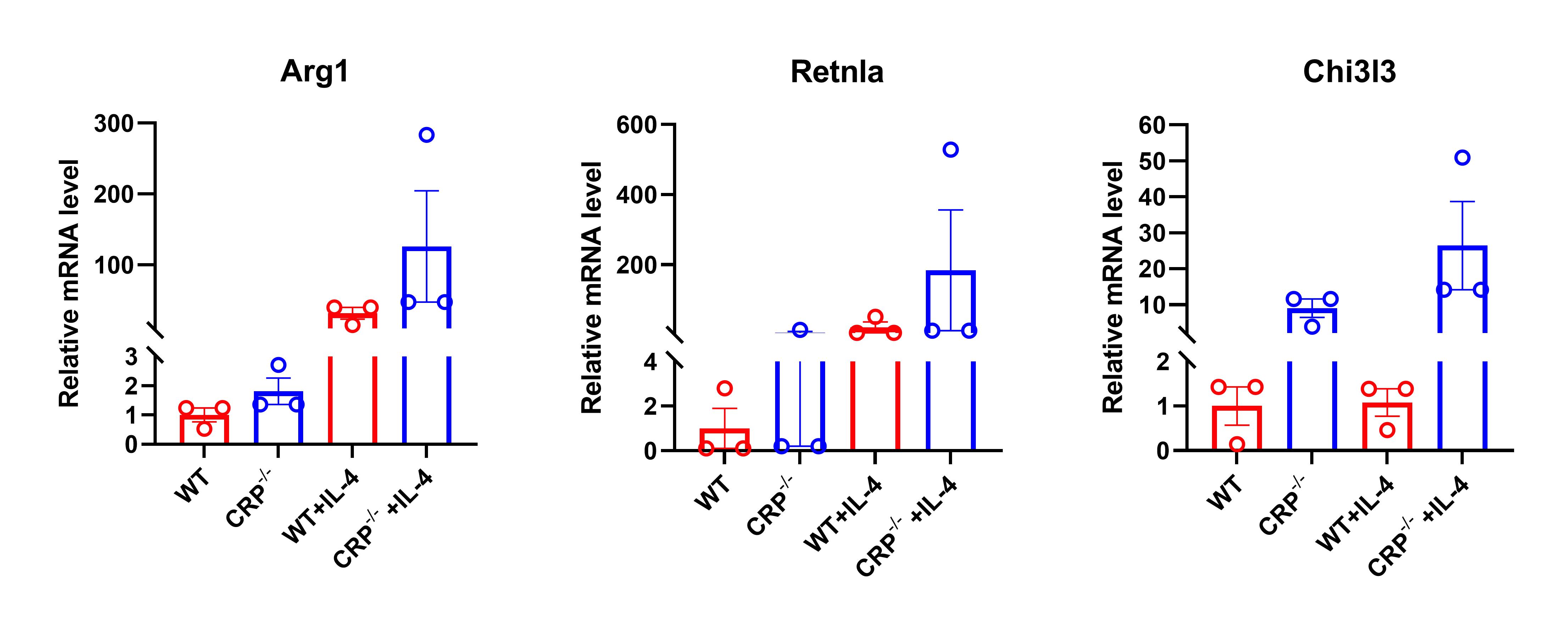

Supplement: Supplementary Figure 1 — The effect of CRP deficiency in IL-4 induced M2 polarization in cultured macrophages. Peritoneal macrophages from non- WT and CRP-/- mice underwent the alternative activation (conventionally known as M2 polarization) in the presence of IL-4 (10 ng/ml) or vehicle alone. The mRNA expression levels of indicated M2 macrophage marker genes were measured by using qRT-PCR. Two-way ANOVA followed two group comparison, no significant influence of CRP deficiency on either M2 maker mRNA levels, n=3 biological repeats for each group. [file Image_1.jpeg]
